# Comprehensive Genomic Profiling of Cell-Free Circulating Tumor DNA Detects Response to Ribociclib Plus Letrozole in a Patient with Metastatic Breast Cancer

**DOI:** 10.3390/biom12121818

**Published:** 2022-12-06

**Authors:** Catarina Silveira, Ana Carla Sousa, Patrícia Corredeira, Marta Martins, Ana Rita Sousa, Arnaud Da Cruz Paula, Pier Selenica, David N. Brown, Mahdi Golkaram, Shannon Kaplan, Shile Zhang, Li Liu, Britta Weigelt, Jorge S. Reis-Filho, Luís Costa, Maria Carmo-Fonseca

**Affiliations:** 1 GenoMed—Diagnósticos de Medicina Molecular, S.A., Avenida Professor Egas Moniz, 1649-028 Lisboa, Portugal; 2 Instituto de Medicina Molecular João Lobo Antunes, Faculdade de Medicina da Universidade de Lisboa, Avenida Professor Egas Moniz, 1649-028 Lisboa, Portugal; 3 Serviço de Oncologia Médica, Hospital de Santa Maria, Centro Hospitalar Lisboa Norte, Centro Académico de Medicina de Lisboa, Avenida Professor Egas Moniz, 1649-035 Lisboa, Portugal; 4 Department of Pathology and Laboratory Medicine, Memorial Sloan Kettering Cancer Center, 1275 York Avenue, New York, NY 10065, USA; 5 Illumina Inc., 5200 Illumina Way, San Diego, CA 92122, USA

**Keywords:** liquid biopsy, circulating cell-free DNA, metastatic breast cancer, ribociclib plus letrozole

## Abstract

Analysis of cell-free circulating tumor DNA obtained by liquid biopsy is a non-invasive approach that may provide clinically actionable information when conventional tissue biopsy is inaccessible or infeasible. Here, we followed a patient with hormone receptor-positive and human epidermal growth factor receptor (HER) 2-negative breast cancer who developed bone metastases seven years after mastectomy. We analyzed circulating cell-free DNA (cfDNA) extracted from plasma using high-depth massively parallel sequencing targeting 468 cancer-associated genes, and we identified a clonal hotspot missense mutation in the *PIK3CA* gene (3:178952085, A > G, H1047R) and amplification of the *CCND1* gene. Whole-exome sequencing revealed that both alterations were present in the primary tumor. After treatment with ribociclib plus letrozole, the genetic abnormalities were no longer detected in cfDNA. These results underscore the clinical utility of combining liquid biopsy and comprehensive genomic profiling to monitor treatment response in patients with metastasized breast cancer.

## 1. Introduction

The development of metastases is a major cause of death in cancer patients [1]. While many tumors can be cured when detected early, once metastasis forms, most cancers become incurable [2]. In the case of breast cancer, metastasis can be found in the lungs, liver and brain, in addition to lymph nodes, but the bone is the most-affected site [3]. Survival outcomes of breast cancer patients differ depending on metastatic sites, with bone metastasis associated with the best prognosis, and brain metastasis associated with the worst survival [4].

Although metastatic disease can be present at diagnosis, most often, metastases are detected months or years following initial diagnosis and treatment. In breast cancer, metastatic recurrence has been reported ranging from months to decades after surgery [5]. Such long-term relapse of disease in a patient who was clinically asymptomatic can be attributed to cancer dormancy [6]. This phenomenon is likely caused by cancer cells that escaped from the primary tumor and disseminated throughout the body [7]. For unknown reasons, these micrometastases remain ‘dormant’ in secondary sites and evade anti-cancer therapies [8]. How the body controls the proliferation of microclusters of disseminated tumor cells and why they occasionally transform into growing metastases is unclear [8].

The emergence of targeted therapies to treat metastatic breast cancer is extending life expectancy [9,10]. In parallel, the development of non-invasive biomarker assays based on liquid biopsy promises to enable the early detection of breast cancer relapse. Recent studies showed that genomic alterations identified in the primary tumor of breast cancer patients could be detected in circulating cell-free DNA (cfDNA) analyzed from plasma samples collected approximately 10 months prior to clinical or radiological relapse [11,12]. Thus, liquid biopsy may contribute to initiating treatment of metastatic disease at an earlier stage. In this regard, a meta-analysis study concluded that the earlier detection of all breast cancer recurrences would result in an absolute reduction in mortality of 17–28% [13].

Liquid biopsy tests might not only identify recurrence early, but also inform the selection of optimal treatment strategies [8]. Indeed, the clonal heterogeneity of tumor cells limits efficacy and duration of response to targeted treatments in metastatic cancer [14]. The analysis of cfDNA in plasma may be sufficient to identify somatic alterations contributed by distinct metastases, potentially circumventing the problem of lacking access to multiple metastatic tumor tissue samples due to associated risks and costs [15].

The detection of circulating cell-free tumor DNA (ctDNA) requires very sensitive molecular assays. Although PCR-based technologies, including droplet digital PCR, are powerful methods for the accurate quantification of a scarce amount of circulating nucleic acids in plasma, they can only test a few mutations per assay [16]. In contrast, recent developments in massively parallel sequencing (also known as next generation sequencing) technologies allow for the comprehensive genomic profiling of entire exonic regions of hundreds of cancer-relevant genes, identifying base substitutions, insertions or deletions, copy number alterations, and gene rearrangements [17,18,19].

Here, we used an established tumor-normal massively parallel sequencing assay [20,21] to characterize the genetic alterations present in the cfDNA extracted from the plasma of a breast cancer patient who developed bone metastases. We identified two DNA abnormalities that were already present in the primary tumor genomic DNA. After treatment with ribociclib plus letrozole, the patient showed a significant clinical improvement, and the two genetic alterations were no longer detected in the plasma.

## 2. Materials and Methods

### 2.1. cfDNA Extraction from Blood Samples

Within 1 to 2 h after blood collection in EDTA tubes, whole blood was centrifuged at 1600*g* for 10 min at room temperature. Then, the supernatant was transferred into falcon tubes, wasting about 5 mm of plasma to avoid buffy-coat disturbance. Next, the plasma samples were centrifuged at 3000*g* for 10 min at room temperature. This high g-force centrifugation step removes cellular debris and thereby reduces the amount of cellular or genomic DNA and RNA in the sample. After this step, the supernatants were collected into microtubes (2 or 5 mL), without disturbing the pellet containing cell debris. Plasma samples were frozen and stored at −80 °C.

For cfDNA extraction, 4 mL of plasma were thawed. The cfDNA was purified using QiAamp^®^ MinElute^®^ ccfDNA kit from Qiagen, according to the manufacturer’s instructions. The cfDNA was eluted in 50 µL of ultra-clean water and quantified using the Qubit^®^ 3.0 Fluorometer (Invitrogen, Life Technologies, Carlsbad, CA, USA) with Qubit^®^ dsDNA HS Assay kit (Invitrogen, Life Technologies), according to the manufacturer’s protocol. Sample quality was assessed using High Sensitivity D1000 ScreenTape (TapeStation, Agilent Technologies, Santa Clara, CA, USA), according to the manufacturer’s instructions. The purified cfDNA was stored at −80 °C.

To assess quality of extracted cfDNA, samples were analyzed using a fragment analyzer (TapeStation 4200, Agilent Technologies). This assay uses a fluorescently stained double-stranded DNA and separates nucleic acids by means of electrophoresis. The TapeStation Analysis software automatically determines size, quantity, and purity of each sample. The size determination is based on a known ladder with specific sizing standards. The known concentration of the upper marker is used to determine concentration values.

### 2.2. Genomic DNA Extraction from Blood Samples

Genomic DNA (gDNA) was extracted from the buffy-coat of the blood sample collected before enrollment in the clinical trial. After removal of plasma, a red blood cell lysis buffer (in-house solution) was added and incubated for 10 min at 4 °C. Then, the sample was centrifuged at 250*g* for 10 min. The supernatant was discarded and the pellet containing white blood cells was washed with PBS 1x (Sigma, St. Louis, MI, USA). After centrifugation at 250*g* for 10 min, the supernatant was discarded, and the pellet was resuspended in 1 mL of PBS 1x (Sigma) and again centrifuged at 250*g* for 10 min. The supernatant was discarded, and the dry pellet was stored at −80 °C.

DNA was extracted using a QIAmp^®^ Blood mini kit from Qiagen, according to the manufacturer’s instructions. The DNA was quantified using Qubit^®^ 3.0 Fluorometer (Invitrogen, Life Technologies) with the Qubit^®^ dsDNA HS Assay kit (Invitrogen, Life Technologies), according to the manufacturer’s protocol.

### 2.3. Sequencing and Analysis of cfDNA and gDNA Extracted from Blood Samples

Both the cfDNA and matched normal gDNA were subjected to massively parallel sequencing using an established tumor-normal assay (Memorial Sloan Kettering-Integrated Mutation Profiling of Actionable Cancer Targets; MSK-IMPACT) that targets 468 cancer-related genes [20,21]. Sequencing data were processed and analyzed as previously reported [22,23,24]. Briefly, reads were aligned to the reference human genome GRCh37 using the Burrows–Wheeler Aligner (v0.7.15) [25]. Local realignment, duplicate removal, and base quality recalibration were performed using the Genome Analysis Toolkit (v3.7) [26]. Somatic single-nucleotide variants (SNVs) were detected by MuTect (v1.0) [27], and small insertions and deletions (indels) were detected using a combination of Strelka (v2.0.15) [28], VarScan2 (v2.3.7) [29], Lancet (v1.0.0) [30], Scalpel (v0.5.3) [31], and Platypus [32]. Pathogenic mutations were defined as variants that were deleterious and/or mutational hotspots. Allele-specific copy number alterations (CNAs) and loss of heterozygosity (LOH) were defined using FACETS [33], as previously described [23,34]. The fraction of the genome altered was computed from the CNAs obtained from FACETS. The cancer cell fraction of each mutation was determined using ABSOLUTE (v1.0.6) [35], as previously described [22,23,34].

### 2.4. Sequencing and Analysis of Primary Tumor Genomic DNA

DNA was extracted from the FFPE primary tumor sample, and Illumina DNA Prep with Enrichment was used for generating whole-exome sequencing libraries, with 40 ng input DNA, as previously described [36]. In brief, following quantification with Qubit^®^ dsDNA High Sensitivity assay, four libraries were pooled for enrichment (4-plex) such that 500 ng of each library was used. Target enrichment was performed using IDT xGen Exome Research Panel. A single hybridization was done overnight at 58 °C, with 12 cycles of post-enrichment PCR. Libraries were quantified by Qubit^®^ dsDNA High Sensitivity assay, normalized, and pooled. Samples were sequenced with 151 bp paired-end reads on the NovaSeq 6000 S4 flow cell using the XP workflow for individual lane loading.

Whole-exome sequencing data were processed as previously described [36]. An unpaired normal sample was used to perform variant calling. All germline variants observed in a database curated in-house, which includes the most common germline variants present in dbSNP [37], were removed. Copy number changes were estimated as previously described [36]. Tumor purity and ploidy were estimated using Sequenza 2.1, and sciClone 1.1 was used for clonality estimation [36].

## 3. Results

### 3.1. Clinical Case

The patient is a woman who was first admitted to hospital in June 2009, at the age of 34 years. She presented with a palpable mass (4 × 4 cm) in the upper outer quadrant of the right breast, with no skin alterations, and an axillary lymphadenopathy on the right side (0.5 × 1.0 cm). A diagnostic mammogram and breast ultrasound showed a hypoechoic area in the upper outer quadrant, with irregular borders and 18 mm of diameter. A microbiopsy was performed that revealed an invasive ductal carcinoma of not otherwise specified (NOS) that was estrogen receptor-positive (ER+), progesterone receptor-positive (PR+), human epidermal growth factor receptor 2-negative (HER2-). p53 was normal as detected by immunohistochemistry.

The patient started neoadjuvant chemotherapy (CTX) with doxorubicin together with cyclophosphamide. After the 5th cycle of treatment, a computed tomography (CT) scan of the abdomen and pelvis revealed a tumor in the right ovary (5 cm). In December 2009, the patient was subjected to a breast conservative surgery with axillary lymph node dissection, and a right salpingo-oophorectomy. The histological exam revealed a residual invasive ductal carcinoma NOS in multiple areas with positive margins, an axillary lymph node metastasis of the same type, and a mature cystic teratoma of the ovary (6 cm).

In January 2010, the patient underwent a mastectomy. Post-surgical treatment was adjuvant CTX with docetaxel and, subsequently, hormonal therapy with goserelin and tamoxifen. The patient also underwent adjuvant radiotherapy. Analysis of genomic DNA extracted from a blood sample revealed no pathogenic germline mutations in the *BRCA1* and *BRCA2* genes.

In September 2017, the patient presented with knee pain. A chest-abdomen-pelvis CT scan showed multiple lytic bone lesions, with soft-tissue involvement in the right iliac (Table 1). Lytic bone lesions were also detected in lumbar vertebral bodies (Table 1). The patient enrolled in an open-label clinical phase 3b trial with ribociclib combined with letrozole (CompLEEment-1, NCT02941926). A considerable clinical improvement was observed after treatment, including a decrease in pain score and partial remission of the target bone lesion in the pelvis at the CT scan (Table 1). In November 2018, the patient was treated with denosumab (120 mg at 4-week intervals). A partial response of the target and non-target lesions was observed until the 24th cycle (Table 1).

### 3.2. Genomic Profiling of Plasma cfDNA

Blood samples were collected before (Pre-cfDNA) and after (Post-cfDNA) the patient enrolled in the clinical trial, and cfDNA was extracted as indicated in Table 2.

**Figure 1:**
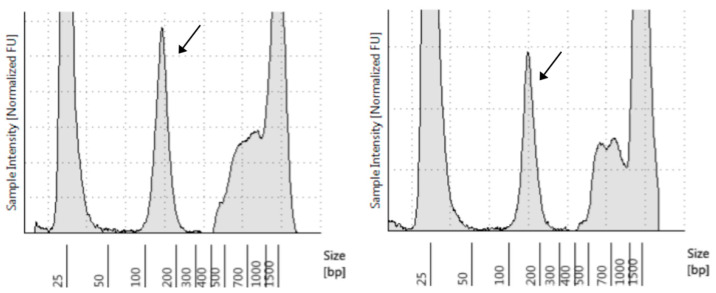
TapeStation analysis of DNA extracted from plasma collected before (**left**) and after (**right**) treatment with ribociclib plus letrozole. Arrows indicate cfDNA fragments (with a peak at ~150 bp).

A gDNA sample was additionally extracted from the buffy-coat obtained from the blood collected in September 2017. In both cfDNA samples, we detected cfDNA fragments with sizes ranging between 70 to 200 base pairs (bp), with a peak at approximately 150 bp (Figure 1).

**Figure 2:**
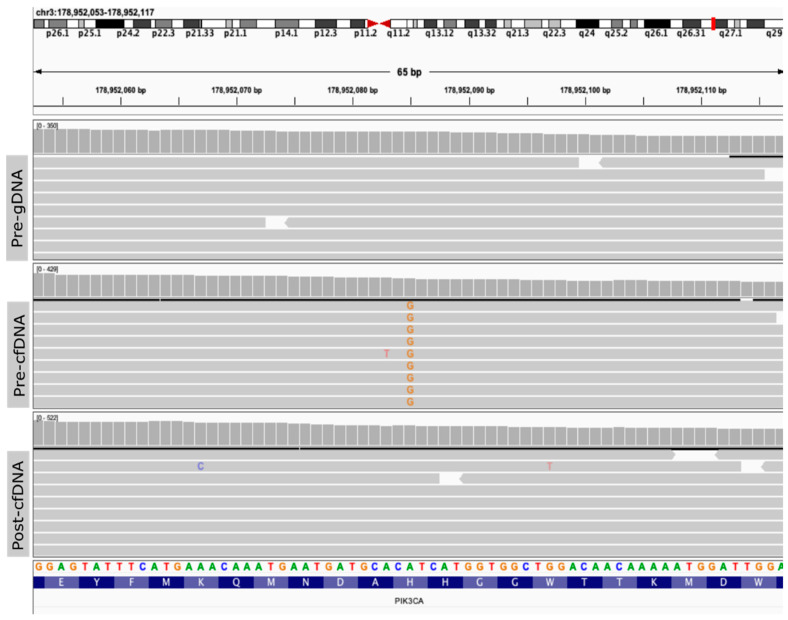
Integrative Genomics Viewer (IGV) screenshot of the *PIK3CA* variant detected in cell-free DNA using targeted massively parallel sequencing. The top panel depicts the sequencing reads of the genomic DNA from buffy-coat collected before treatment (Pre-gDNA). The middle and bottom panels depict the sequencing reads of the cell-free (cf)DNA collected before (Pre-cfDNA) and after (Post-cfDNA) treatment with ribociclib plus letrozole. *PIK3CA* hotspot mutation (3:178952085, A > G, H1047R) was detected only in cfDNA before treatment (middle, alternate alleles are shown in orange).

**Figure 3:**
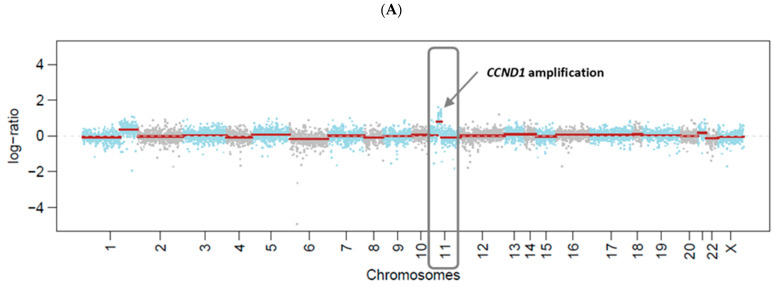

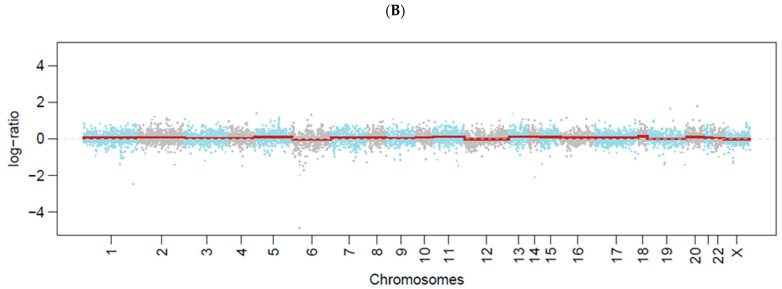
Copy number analysis of cell-free DNA (cfDNA) extracted from plasma collected before (**A**) and after (**B**) treatment with ribociclib plus letrozole. Amplification of the *CCND1* gene in chromosome 11 (arrow) was detected at baseline before treatment, but not after therapy.

Pre-cfDNA was subjected to massively parallel sequencing using the MSK-IMPACT assay that targets 468 cancer-related genes, detecting all protein-coding mutations, copy number alterations, and selected promoter mutations and structural rearrangements [20,21]. The sequencing panel includes oncogenes, tumor suppressor genes, and members of pathways deemed actionable by targeted therapies, and are recurrently altered in cancer. This sequencing assay has been employed for the study of >25,000 tumors [38] as well as cfDNA [24]. Two genomic alterations were detected: a missense mutation in the *PIK3CA* gene (3q26.32, Figure 2) and an amplification of the *CCND1* gene (11q13.3, Figure 3). The *PIK3CA* hotspot mutation (3:178952085, A > G, H1047R) was present at a variant allele frequency (VAF) of 0.14 (28 out of 200 reads). This variant was present in an estimated cancer cell fraction (CCF) of 0.97, indicating that the variant is likely clonal. The matched normal gDNA sample had a coverage of 199 reads at this position (3:178952085), and no altered reads were detected.

The presence of both molecular alterations was confirmed in gDNA from the primary tumor. In the primary tumor tissue, the hotspot mutation in *PIK3CA* (H1047R) was present with an estimated CCF of 1. This mutation was detected with an estimated purity of 0.34 and a ploidy of 1.8, with a normal allelic depth of 392 and a tumor allelic depth of 93 (VAF = 0.2). Additionally, amplification of the *CCND1* gene was observed, with an estimated fold change of 1.25. No other molecular changes were identified.

The analysis of cfDNA after treatment (Post-cfDNA) did not detect either of the two alterations. In the *PIK3CA* gene, we identified 266 reads covering the position of interest (3:178952085), and none presented this variant (Figure 2). Moreover, amplification of the *CCND1* gene was no longer observed (Figure 3). Thus, the results in cfDNA mirror the clinical response.

## 4. Discussion

This study highlights the utility of cfDNA analysis for therapy monitoring in metastatic breast cancer patients. Our results are consistent with previous reports indicating that circulating tumor DNA can be used as surrogate marker of treatment outcome [15,39]. Recently, genotyping cfDNA in plasma samples from patients in the randomized phase III PALOMA-3 study of CDK4/6 inhibitor palbociclib and fulvestrant for women with advanced ER+ breast cancer showed that a reduction in the levels of mutant *PIK3CA* DNA detected in circulation correlated with improved progression-free survival (PFS) after treatment [40]. Similarly, patients with ER+ advanced metastatic breast cancer enrolled in the phase I/II randomized BEECH trial (paclitaxel plus placebo versus paclitaxel plus AKT inhibitor capivasertib) with decreased levels of mutant cfDNA detected in plasma after 4 weeks of treatment had substantially improved PFS [41].

Using massively parallel sequencing to analyze cfDNA in the patient plasma before treatment, we detected the *PIK3CA* hotspot mutation H1047R. *PIK3CA* is one of the two most frequently mutated genes in breast cancers, occurring in 30–40% of cases, and H1047R is the most common mutation in this gene [42,43]. The *PIK3CA* gene encodes the catalytic subunit of phosphatidylinositol 3-kinase (PI3K), and the H1047R mutation induces gain of enzymatic function, allowing PI3K to signal without regulation and triggering oncogenic properties [44,45]. When present, *PIK3CA* mutations are typically found in both the primary tumor and in the relapsed/metastatic tissue [46]. Consistent with the finding that *PIK3CA* mutations are predominantly truncal events in breast cancer, we identified the H1047R mutation to be clonal and likely early occurrence in tumor evolution. Notably, a previous study showed that truncal mutations in *PIK3CA* detected by liquid biopsy predicted sensitivity to palbociclib, whereas sub clonal mutations were weak predictors of outcome [40]. More recently, the sequencing of circulating tumor DNA in patients enrolled in the phase III MONALEESA-7 trial revealed a treatment response to endocrine therapy plus ribociclib independent of the *PIK3CA* mutational status [47].

In addition to the *PIK3CA* hotspot mutation, our cfDNA analysis identified the amplification of *CCND1*, an oncogene that encodes the protein cyclin D1. The cyclin dependent kinases 4 and 6 (CDK4/6) form complexes with D-type cyclins that act on the retinoblastoma protein Rb and drive cell cycle progression [48]. *CCND1* amplification leads to increased cyclin D1 expression and inappropriate cyclin D–CDK4/6 activity [49,50], thus promoting sustained cell proliferation, which is one of the hallmarks of cancer [51]. *CCND1* amplification occurs in 10–35% of breast cancers and is typically associated with positive ER status [49,50]. Breast cancer patients with *CCND1* amplification tend to show a poor response to endocrine therapy [50], which may be related to the ability of cyclin D1 to stimulate the growth of estrogen responsive tissues through a CDK-independent mechanism by activating the transcription of ER-regulated genes in the absence of estrogen [52]. However, the clinical benefit after ribociclib and endocrine therapy was observed in advanced ER+/HER2- breast cancer patients with altered *CCND1* [47].

The patient reported in this study was treated with the CDK4/6 inhibitor ribociclib combined with the aromatase inhibitor letrozole. After treatment, the patient had a significant clinical improvement, and no molecular abnormalities were detected by massively parallel sequencing of cfDNA. A drawback of sequencing cfDNA is the problem of false negatives. Indeed, not all cancer cells release their DNA into circulating blood, and the concentration of cell-free tumor DNA in the plasma may be below the sensitivity of available technologies. However, in this case, the cfDNA results mirrored the clinical response. A limitation of our study is that we did not monitor the patient cfDNA prospectively to determine whether detectable genetic alterations could be detected prior to clinical relapse.

Based on the results of recent trials, ribociclib plus letrozole is currently considered the frontline treatment option in postmenopausal patients with advanced ER+/PR+/HER2- breast cancer [53,54]. Although these trials showed a consistent overall survival benefit, future studies are needed to stratify drug response according to subgroups defined by patient and disease characteristics. In this regard, comprehensive profiling of cfDNA isolated from plasma samples may contribute a real-time assessment of driver and actionable mutations and their clonal evolution in response to treatment.

## Figures and Tables

**Table 1:** Lesions follow-up according to the RECIST 1.1 criteria.

Target Lesion	Lytic Bone Lesions, Right Iliac with Soft-Tissue Involvement							
**Follow-up date**	19 December 2017	13 March 2018	5 June 2018	27 September 2018	4 December 2018	11 March 2019	4 June 2019	29 August 2019
**Size**	81 mm	58 mm	57 mm	54 mm	48 mm	47 mm	46 mm	42 mm
**Non-target lesion**	**Lytic bone lesions, lumbar vertebral bodies**							
**Follow-up date**	19 December 2017	13 March 2018	5 June 2018	27 September 2018	4 December 2018	11 March 2019	4 June 2019	29 August 2019
**Number**	Multiple	Multiple	Stable	Stable	Stable	Stable	Stable	Stable

**Table 2:** Blood and plasma sample details.

Sample ID	Collection Date	Sample Type	Sample Concentration(ng/uL)	Sample Volume(µL)
**Pre gDNA**	18 September 2017	Buffy-coat	70.5	40.0
**Pre cfDNA**	18 September 2017	Plasma (4 mL)	0.7	45.0
**Post cfDNA**	25 June 2018	Plasma (4 mL)	0.3	45.0

## Data Availability

Not applicable.
